# Right apical-posterior segmentectomy with abnormal anterior segmental bronchus and artery: a case report

**DOI:** 10.1186/s13019-021-01570-3

**Published:** 2021-07-06

**Authors:** Xining Zhang, Gang Lin, Jian Li

**Affiliations:** Department of thoracic surgery, Peking University First Hospital, Xishiku street 8th, Beijing, 10000 China

**Keywords:** Thoracoscopic segmentectomy, Bronchial variation, Inflation-deflation technique, Lung cancer, Case report

## Abstract

**Background:**

Displaced anterior segmental bronchus and pulmonary artery is extremely rare. A keen knowledge of such variations is required in the field of pulmonary segmentectomy, for unawareness of the structural variation could lead to intra- and postoperative complications.

**Case presentation:**

A 50-year-old female presented to our department with suspected lung adenocarcinoma. Preoperative 3-dimensional computed tomographic bronchography and angiography revealed anterior segmental bronchus and anterior segmental pulmonary artery variation: the anterior segmental bronchus derived from the middle lobe bronchus, accompanied by a distally distributed anterior segmental pulmonary artery branch. A right apical-posterior segmentectomy using inflation-deflation technique was performed successfully.

**Conclusions:**

The keen observation and proper application of modern imaging technology and operative technique could greatly aid segmentectomy, preventing intra- and postoperative complications from happening.

## Introduction

Bronchial variation, which is most commonly seen in right upper lobe [1], encompasses a spectrum of less-than-common distributional patterns of bronchus. There has been case report concerning bronchial variation associated with pulmonary vessel anomalies and incomplete fissure [2]. Unawareness of such anomalies could lead to intraoperative and postoperative complications, especially when using a thoracoscopic approach. There has been a small number of surgical cases reporting apical and posterior bronchial (B1 and B2) abnormalities [3], however, report of segmentectomy involving variation in anterior segmental bronchus (B3) and artery (A3) is still scant.

It is well documented that anomalous bronchus is frequently associated with under-developed fissure [4]. The appropriate approach to the intersegmental fissure is essential to reduce air leakage, minimize postoperative ventilation-perfusion mismatch and lung function loss. The inflation-deflation technique (IDT) [5] plays an important role in identifying the intersegmental plane for a thoracoscopic procedure.

Herein we present a case of thoracoscopic right apical-posterior segmentectomy for lung cancer with displaced anterior segment structures and absence of any fissure in the right upper-middle lobe region in which IDT was applied to delineate proper intersegmental plane.

## Case report

**Fig. 1:**
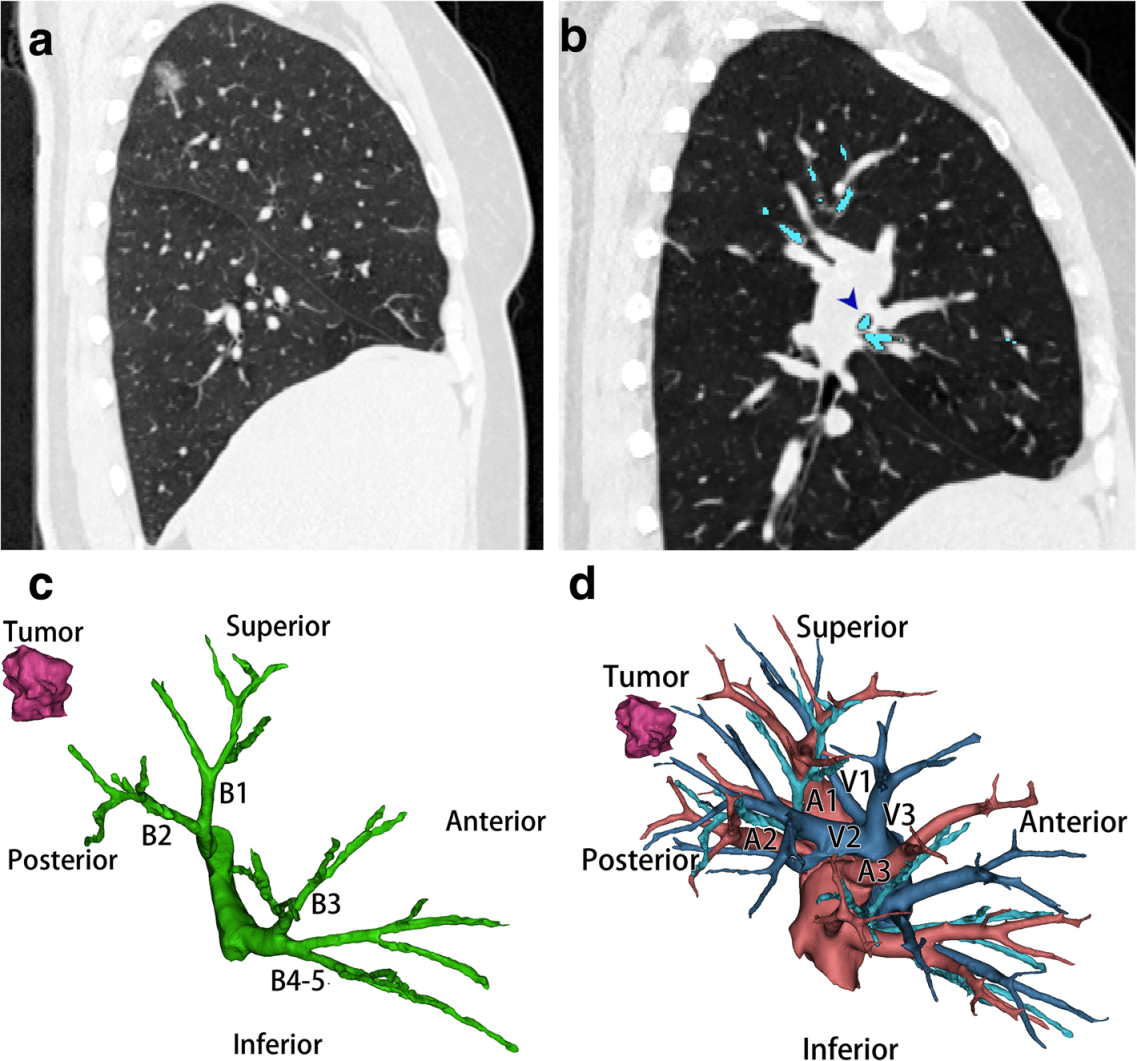
Preoperative computed tomographic and 3-dimentional computed tomographic bronchographic and angiographic images. **a** sagittal image showing a nodule resides in the apical-posterior segment. **b** sagittal image showing the anterior segmental bronchus derives from the middle lobe bronchus (arrow). **c** The distribution of right upper and middle lobe bronchi, noted that the anterior segmental bronchus derived from middle lobe bronchus. **d** The distribution of right upper and middle lobe vessels, noted that the anterior segmental pulmonary artery branched from the pulmonary trunk distally to the branching sites of apical and posterior arteries. The distribution of pulmonary vein is relatively normal

A 50-year-old, otherwise healthy female presented to our department with a part-solid ground-glass nodule. The patient was asymptomatic, and chest CT revealed a part-solid ground-glass nodule of maximum diameter 19*14*19 mm in the posterior segment of the right upper lobe (Fig. 1A). An abnormal right B3 was identified in the Chest CT: the right upper bronchus gave rise to apical and posterior segmental bronchi, and the B3 derived from the right middle lobe bronchus (Fig. 1B); A3 was the third branch of the pulmonary trunk, following apical and posterior branches. Those variations are more clearly displayed in the 3-dimensional computed tomographic bronchography and angiography (3D-CTBA) (Fig. 1C and D). The distribution of pulmonary vein was relatively normal. No fissure was detected in the right upper-middle lobe region. Preoperative examinations include physical examination, electrocardiogram, abdominal ultrasound, hematology examinations, pulmonary function tests, bone scan and echocardiography. All results were normal without evidence of metastasis. According to the eighth edition of TNM staging system, the clinical staging of this patient was stage IA2 (T_1b_N_0_M_0_).

Considering that the nodule was in the posterior segment of the right upper lobe and the variation of anterior segmental bronchus and pulmonary artery, though the pre-operative results (forced expiration volume in 1 s: 2.21 L) did not suggest that a compromised segmentectomy needed to be performed, an intentional dual portal thoracoscopic apico-posterior segmentectomy was deemed the procedure of choice for this patient.

**Fig. 2:**
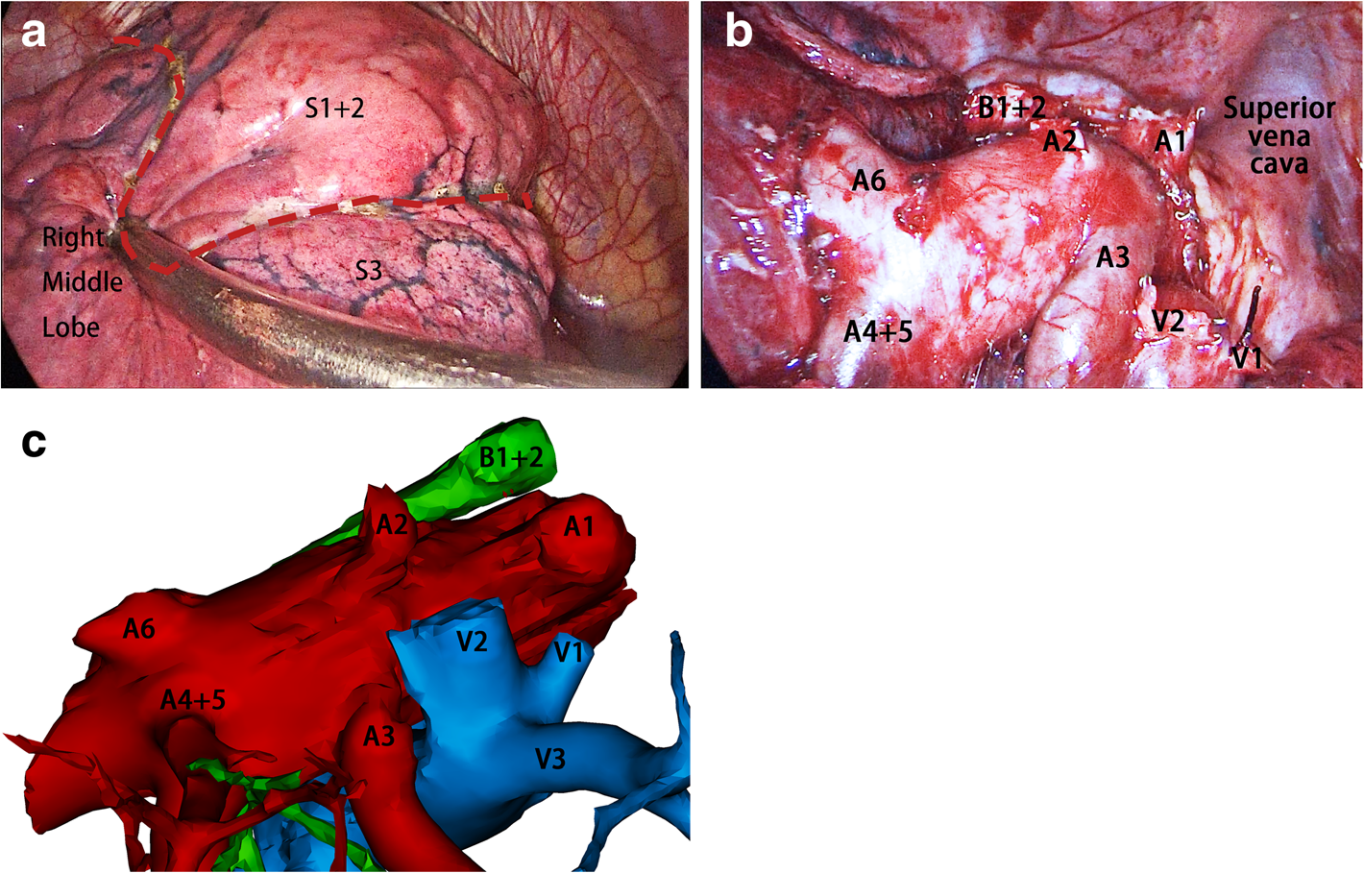
Images of intraoperative findings and simulated 3-dimensional reconstructed hilar structures after apical-posterior segmentectomy. **a** the intersegmental plane between apical and anterior segments after the inflation-deflation technique is applied (red dashed line). **b** the hilar structures after the removal of right apical-posterior segments, showing the spatial relationships of branching and tributary points of bronchi, pulmonary arteries and veins. **c** the simulated 3-dimensional reconstruction graph of hilar structures in a similar view angle of (**b**)

After general anesthesia, the patient was put into left decubitus position. The apical segmental artery and vein were dissected and divided first. The B1 + 2 was then identified, ligated and divided after the peri-bronchial lymph node dissection. With retraction of the right upper lobe caudally, the posterior segmental pulmonary artery was dissected and divided. Finally, the central vein was dissected and divided. IDT was then applied to delineate the boundary between apical-posterior and anterior segments: double lung ventilation was applied temporally after the division of apico-posterior bronchus to inflate the right lung, and isolated left lung ventilation was resumed after the confirmation that right lung was fully inflated, at this time, unlike in the other segments, air in the apico-posterior segment could not be exhaled as the apico-posterior bronchus was divided, this differential deflation creates a easily recognizable intersegmental plane, which was divided using multiple staplers (Fig. 2A). Then the right S1 + 2 was retrieved (Fig. 2B and C). Intraoperative frozen-section pathologic examination for the resected specimen confirmed the diagnosis of Acinar predominant invasive adenocarcinoma without more invasive histologic subtype and spread through air space, a systematic lymph node sampling was then performed. The operation lasted 1 h and 34 min, and the blood loss was less than 50 ml. The postoperative course was uneventful and the patient was discharged on postoperative day 4. Postoperative pathologic examination confirmed the diagnosis of acinar predominant adenocarcinoma. There was no sign of involvement of visceral pleura or lympho-vascular invasion. No metastasis was found in the lymph nodes, the pathologic stage was stage IA2 (T_1b_N_0_M_0_).

## Discussion and conclusion

Two features of our case are of interest here: the identification of displaced B3, which stemmed from right middle lobe bronchus, and the delineation of intersegmental plane using IDT. To our best knowledge, this is the first surgical case in which the intersegmental plane was determined by IDT in a patient with lung cancer with displaced B3 and A3.

Numerous bronchial anomalies have been described previously. According to Yaginuma [1], the prevalence of bronchial variation is 0.76%, and the majority (84.8%) of bronchial abnormality involves right upper lobe region, however, there have been few surgical cases pertinent to B3 variation reported. This variation is considered extremely rare, especially in patients with lung cancer. One similar case was reported by Nakanishi [2], in which they performed a right upper lobectomy in a patient with lung cancer and displaced B3.

Various intersegmental plane detecting methods were developed recently. Generally, the majority of them is in agreement with the “hilum first, fissure last” technique, which is frequently applied when incomplete interlobar fissure is encountered in a lobectomy. Different from Nakanishi et al., who used intravenous indocyanine green (ICG) with fluorescence imaging [2] to recognize intersegmental plane, IDT [5] is routinely performed in our center. IDT is able to distinctly delineate the proper intersegmental plane in about 10 min, bears no risk of anaphylactic reaction and doesn’t require fluorescence imaging.

Evidence has been gathering suggesting the non-inferiority of segmentectomy comparing to lobectomy in early-stage NSCLC provided that margin handling and lymph node harvesting be executed properly [6, 7]. Therefore, the apical-posterior segmentectomy with systemic lymph node sampling was deemed the procedure of choice for this patient. We would also like to recommend performing intraoperative frozen section for the resected specimen to rule out more aggressive subtypes of adenocarcinoma, for segmentectomy may not be adequate for those subtypes [8].

In conclusion, the variations of major pulmonary structures warrant particular attention. An appropriate way to address rare abnormalities is to routinize preoperative 3D-CTBA for segmentectomy and other parenchyma resection with anatomic variation. Surgeons must stay aware of both the common and uncommon patterns of bronchus and pulmonary vessels.

## Data Availability

All data generated or analyzed during this study are included in this published article.

## Abbreviations

B1: Apical segmental bronchus
B2: Posterior segmental bronchus
B3: Anterior segmental bronchus
A3: Anterior segmental pulmonary artery
IDT: Inflation-deflation technique
3D-CTBA: 3-dimensional computed tomography bronchography and angiography

## References

[1] Yaginuma H (2020). Investigation of displaced bronchi using multidetector computed tomography: associated abnormalities of lung lobulations, pulmonary arteries and veins. Gen Thorac Cardiovasc Surg.

[2] Nakanishi K, Kuroda H, Nakada T, Ueno H, Sakakura N (2019). Thoracoscopic lobectomy using indocyanine green fluorescence to detect the interlobar fissure in a patient with displaced B3 and absence of fissure: a case report. Thorac Cancer.

[3] Xu X-f, Chen L, Wu W-b, Zhu Q (2014). Thoracoscopic right posterior Segmentectomy of a patient with anomalous bronchus and pulmonary vein. Ann Thorac Surg.

[4] Hiroshi Y, Ken-Ichiro T, Masashi U (2021). Right top pulmonary veins associated with lung incomplete fissure and displaced bronchus: a retrospective study using multidetector computed tomography. Gen Thorac Cardiovasc Surg.

[5] Chen L, Wu W (2016). The Main technical points of thoracoscopic anatomical lung segment resection. Zhongguo Fei Ai Za Zhi.

[6] Winckelmans T, Decaluwé H, De Leyn P, Van Raemdonck D (2020). Segmentectomy or lobectomy for early-stage non-small-cell lung cancer: a systematic review and meta-analysis. Eur J Cardiothorac Surg.

[7] Lin G, Liu H, Li J (2019). Lobectomy versus sub-lobar resection in patients with stage IA right middle lobe non-small cell lung cancer: a propensity score matched analysis. J Thorac Dis.

[8] Nitadori J, Bograd AJ, Kadota K, Sima CS, Rizk NP, Morales EA, Rusch VW, Travis WD, Adusumilli PS (2013). Impact of micropapillary histologic subtype in selecting limited resection vs lobectomy for lung adenocarcinoma of 2cm or smaller. J Natl Cancer Inst.

